# Non-Fourier Bioheat Transfer Analysis in Brain Tissue During Interstitial Laser Ablation: Analysis of Multiple Influential Factors

**DOI:** 10.1007/s10439-023-03433-5

**Published:** 2024-01-18

**Authors:** Sundeep Singh, Leonardo Bianchi, Sanzhar Korganbayev, Pouya Namakshenas, Roderick Melnik, Paola Saccomandi

**Affiliations:** 1 Faculty of Sustainable Design Engineering, University of Prince Edward Island, Charlottetown, PE C1A 4P3 Canada; 2 Department of Mechanical Engineering, Politecnico di Milano, 20156 Milan, Italy; 3 MS2Discovery Interdisciplinary Research Institute, Wilfrid Laurier University, Waterloo, ON Canada

**Keywords:** Bioheat transfer, Brain, Laser ablation, Mathematical modeling, Non-Fourier heat transfer, Thermal therapy

## Abstract

This work presents the dual-phase lag-based non-Fourier bioheat transfer model of brain tissue subjected to interstitial laser ablation. The finite element method has been utilized to predict the brain tissue's temperature distributions and ablation volumes. A sensitivity analysis has been conducted to quantify the effect of variations in the input laser power, treatment time, laser fiber diameter, laser wavelength, and non-Fourier phase lags. Notably, in this work, the temperature-dependent thermal properties of brain tissue have been considered. The developed model has been validated by comparing the temperature obtained from the numerical and ex vivo brain tissue during interstitial laser ablation. The ex vivo brain model has been further extended to in vivo settings by incorporating the blood perfusion effects. The results of the systematic analysis highlight the importance of considering temperature-dependent thermal properties of the brain tissue, non-Fourier behavior, and microvascular perfusion effects in the computational models for accurate predictions of the treatment outcomes during interstitial laser ablation, thereby minimizing the damage to surrounding healthy tissue. The developed model and parametric analysis reported in this study would assist in a more accurate and precise prediction of the temperature distribution, thus allowing to optimize the thermal dosage during laser therapy in the brain.

## Introduction

The application of minimally invasive thermal ablation using radiofrequency, microwave, laser, ultrasound, and cryoablation has been widely explored in clinical practices for treating different types of tumors. These therapies aim to attain local tissue necrosis by applying extreme temperatures in a focal zone in and around the tumor for a short time (i.e., less than 15 min circa) [1]. Apart from being less invasive (i.e., better cosmesis) compared to open surgery, these thermal ablative modalities also result in reduced cost, increased preservation of surrounding tissue, shorter hospitalization times, and lower morbidity [2]. Laser applicators have a significantly smaller diameter than radiofrequency, microwave, and cryoablation applicators. Owing to this, laser therapies can serve as one of the least invasive thermal ablative modalities, especially in high-risk or complex technical access [3–6]. In general, two types of laser delivery methods have been utilized in the past. One is the most commonly used method of directly focusing the collimated laser beam on the skin for treating subsurface tumors. Another method is known as laser interstitial thermal therapy, whereby the diffusing laser fiber applicator is inserted within the target tissue utilizing image guidance modality for treating deep-seated tumors. The treatment outcomes of the laser ablation are significantly dependent on the irradiation time, intensity, wavelength, and spot size of the laser, as well as the optical and thermal properties of the tissue [7, 8].

During interstitial laser ablation, irreversible damage to the biological tissue is attained by virtue of the photothermal conversion of near-infrared light into heat. The depth of light penetration and the amount of energy delivered depends on the absorption, transmission, scattering, and refraction of laser light within the biological tissue [9]. It is only the absorbed energy that can produce therapeutic effects, which are further affected by the amount and type of chromophores (generally, water and hemoglobin) present within the tissue [4, 8, 10]. Interstitial laser ablation has shown promising results and has been extensively used for treating tumors in the liver, prostate, lung, pancreas, breast, and brain [6, 10–21]. In particular, there has been a dramatic increase in the number of interstitial laser ablation studies focused on treating intracranial tumors in the last decade. Accordingly, interstitial laser ablation has matured and gained prominence for treating glioblastoma (GBM), brain metastases, gliomas, pediatric brain tumors, radiation necrosis, and epilepsy [4, 7, 12, 22–24]. However, maintaining the precise control of the interstitial laser ablation to localized focal zones and thereby mitigating any chances of unintended damage to the surrounding healthy tissue, still remains the major challenge.

Computational modeling and simulation could provide a reasonable route to overcome some of these interstitial laser ablation limitations by better understanding the intrinsic and extrinsic factors that affect heat transfer during light-tissue interactions within the biological tissue. Computational models can also serve as a viable alternative to in vivo experiments. They can be easily extended to more realistic scenarios by integrating and incorporating patient-specific models and properties. These numerical models can also assist in rapid and low-cost evaluation and optimization of the laser applicator design and systems. Significant advances have been reported in these thermal ablative models in the past decade to reach a stage where these models can be readily integrated into the hospital workflow, assisting clinicians by providing *a priori* information on the optimized treatment outcomes for individual patient settings. Most computer models for interstitial laser ablation to date assume Fourier’s law based Pennes’ bioheat transfer equation to conduct heat transfer analysis within biological tissues [10, 12, 16, 20]. These models consider infinitely fast propagation of thermal signals, i.e., any thermal disturbance in the medium will be felt instantaneously throughout the medium. Such assumptions are justified in most practical scenarios but are heavily criticized for biological tissues. To address this issue and to more accurately model the heat transfer analysis in biological tissues, the lagging behavior associated with the finite speed of thermal signals through non-Fourier effects should be considered.

In this study, we report a non-Fourier heat transfer model of interstitial laser ablation of the brain tissue. Importantly, we have used the temperature-dependent thermal properties acquired from ex vivo brain tissue, as previously quantified and reported by our group. We have conducted parametric studies to evaluate the influence of laser fiber diameter, input power, treatment time, laser wavelength, and thermal relaxation times on the temperature distribution during interstitial laser ablation of the brain. The developed numerical model fidelity and integrity have also been evaluated by comparing the results obtained from the numerical studies with those obtained from the ex vivo experimental studies performed on the calf brain tissue.

## Materials and Methods

### Non-Fourier Heat Transfer Model of Interstitial Laser Ablation of the Brain Tissue

**Fig. 1:**
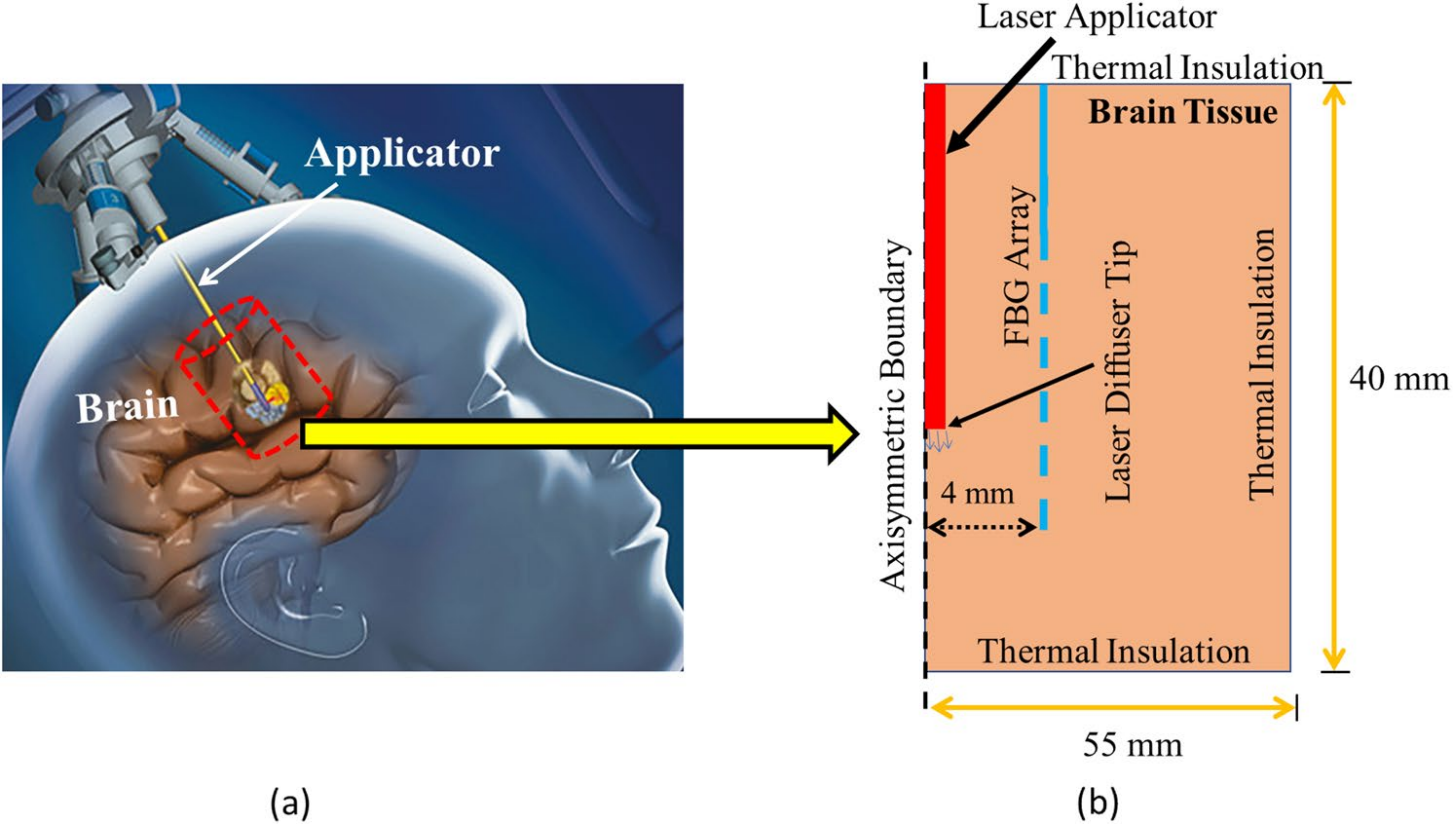
**a** Representation of interstitial laser ablation in the brain whereby an optical fiber, attached to the laser light system, is passed through a burr hole to the desired depth using image-guided modalities, and the laser light is interstitially delivered to heat the tissue (adapted from [4] under the terms of the Creative Commons CC BY License for an open access article). **b** Schematic of the axisymmetric model of the brain tissue derived from the selected cylindrical control volume, also highlighting the location of the fiber Bragg grating (FBG) sensor

A two-dimensional axisymmetric computational domain of homogeneous and isotropic brain tissue subjected to interstitial laser ablation has been presented in Fig. 1. Different heat transfer models are available in the literature to study the complex heat transfer phenomena within biological tissue during thermal ablation. Among these bioheat transfer models, Pennes’s model is the most widely used model for predicting temperature distribution owing to its simplicity, computational efficiency, and effectiveness [7, 8, 10]. Notably, the bioheat transfer model is based on the classical Fourier’s law, which assumes the infinite speed of heat propagation and relates the heat flux with the temperature gradient as [25]:

1 $$q(\vec{r},\;t) = - k\nabla T(\vec{r},\;t),$$

where *q* is the heat flux, *k* is thermal conductivity, and *T* (*r*, *t*) is the temperature at point* r* in time *t*.

The generalized bioheat transfer equation (incorporating water vaporization) in the Cartesian coordinate system is represented as [7, 16, 26, 27]:

2 $$\rho c_{eff} \frac{\partial T}{{\partial t}} = \nabla (k\nabla T) - \omega_{b} \rho_{b} c_{b} (T - T_{b} ) + Q_{met} + Q_{laser} ,$$

where the product *ρc*_*eff*_ (J/(m^3^·K)﻿) is the effective volumetric heat capacity of tissue accounting vaporization computed using Eq. 3, *k* (W/(m·K)) is the thermal conductivity of tissue computed using Eq. 6, *T* (K) is the tissue temperature that needs to be computed from Eq. 2, *ω*_*b*_ (s^−1^) is the blood perfusion rate computed from Eq. 7, *ρ*_*b*_ is the density of blood (= 1050 kg/m^3^) [28], *c*_*b*_ is the specific heat of blood (= 3617 J/(kg·K)) [28], *T*_*b*_ is the temperature of blood (= 37 °C), *Q*_*met*_ (W/m^3^) is the metabolic heat source, which is ignored owing to the insignificant contribution as compared to other heat sources, and* Q*_*laser*_ (W/m^3^) represents the external laser heat source computed using Eq. 9.

The effective volumetric heat capacity (*ρc*_*eff*_), which accounts for the abrupt increase in the thermal capacity of the biological tissue due to the vaporization of water content as the tissue temperature approaches 100 °C, is given as [16, 26, 29]:

3 $$\rho c_{eff} = \rho c - \alpha \frac{\partial W}{{\partial T}} = \rho c - \alpha W^{\prime}_{T} ,$$

where the product *ρc* is the volumetric heat capacity of tissue (J/(m^3^·K)﻿), α is the latent heat of vaporization of water, and *W* is the tissue water content (kg/m^3^) that is a function of temperature (K) and is given as [16, 26, 29]:

4 $$W(T) = 0.778 \times \left| {\begin{array}{*{20}c} {1 - \exp \left( {\frac{T - 106}{{3.42}}} \right)} & {T \le 103\,^{ \circ } {\text{C}},} \\ {0.03713 \cdot T^{3} - 11.47 \cdot T^{2} + 1182 \cdot T - 40582} & {103\,^{ \circ } {\text{C}} < T \le 104\,^{ \circ } {\text{C}}} \\ {\exp \left( { - \frac{T - 80}{{34.37}}} \right)\,} & {T > 104\,^{ \circ } {\text{C}}.} \\ \end{array} } \right.,$$

The formulation details of Eqs. (3)–(4) has been provided in our group’s previous study in [27]. It is worth mentioning that, in this numerical study, we have used the temperature-dependent thermal properties of the ex vivo calf brain tissue, previously reported by our group in [30]. The volumetric heat capacity (J/(m^3^·K)﻿) has been found to be varying exponentially as a function of temperature, as:

5 $$\rho c = 3.732 \times 10^{6} + 9.530 \times 10^{5} \exp (0.240 \cdot T).$$

This exponential function of volumetric heat capacity was substituted in Eq. (3), for computing the effective volumetric heat capacity incorporating tissue vaporization effects. Similarly, the temperature-dependent thermal conductivity (W/(m·K)) of the ex vivo calf brain tissue, previously quantified by our group in [30], has been used till 100 °C, beyond which we have used a decreasing function of thermal conductivity to account for tissue vaporization [26], and is given as:

6 $$k(T) = \left\{ {\begin{array}{*{20}c} {0.558 + 2.261 \times 10^{ - 9} \exp (0.208 \cdot T)} & {T \le 100\,^{ \circ } {\text{C}},} \\ {55.44 - 0.99701 \cdot T + 4.4988 \times 10^{ - 3} T^{2} } & {T > 100\,^{ \circ } {\text{C}}.} \\ \end{array} } \right.$$

Further, microvascular blood perfusion can significantly affect heat transfer during in vivo interstitial laser ablation of the brain. Here, blood perfusion has been modeled as a temperature-dependent piecewise function, as given by:

7 $$\omega_{b} = \left\{ {\begin{array}{*{20}c} {\omega_{o} } & {T < 60\,^{ \circ } {\text{C}},} \\ 0 & {T \ge 60\,^{ \circ } {\text{C}}.} \\ \end{array} } \right.\,$$

where ω_o_ (equal to 9.745 × 10^−3^ s^−1^ [28]) is the constant perfusion rate in undamaged brain tissue that prevails below the temperature of 60 °C and ceases beyond it [31]. Further, apoptosis and necrosis of cells occur at 60 °C; thus, the ablation volume (*V*) attained during the interstitial laser ablation has been quantified using the isotherm of 60 °C, i.e., the volume of brain tissue having temperature ≥ 60 °C after the prescribed treatment time [31], as:

8 $$V = \left\{ {\begin{array}{*{20}c} 0 & {T < 60\,^{ \circ } {\text{C}},} \\ {\iiint_{T} {dV\,}} & {T \ge 60\,^{ \circ } {\text{C}}.} \\ \end{array} } \right.\,$$

The laser heat source term in the bioheat transfer analysis has been modeled utilizing the Beer–Lambert’s law [12, 16, 26, 32–34], accounting for absorption and scattering phenomena of laser light in brain tissue, as:

9 $$Q_{laser} = \mu_{eff} \cdot I \cdot e^{{ - \mu_{eff} z}} ,$$

where *µ*_*eff*_ is the effective attenuation coefficient (m^−1^) computed using Eq. 11, *z* is the depth of tissue (m), and *I* is the laser irradiation intensity (W/m^2^) given by:

10 $$I = I_{o} \cdot e^{{ - \,\,\frac{{r^{2} }}{{2\sigma^{2} }}}} = \frac{P}{{2\pi \sigma^{2} }} \cdot e^{{ - \,\,\frac{{r^{2} }}{{2\sigma^{2} }}}} ,$$

where *I*_0_ is the maximum irradiation intensity at the center of a 2-D Gaussian profile of laser beam (W/m^2^), *σ* (equal to *r*_*f*_/3, where *r*_*f*_ is the radius of a bare fiber applicator) is the standard deviation of the laser beam profile [16], *r* is the radial distance (m), and *P* is the power of continuous-wave mode laser emitter (W). The two clinically U.S. FDA approved laser interstitial thermal ablation systems currently used in the United States are Visualase (Visualase Inc., Houston, Texas, USA), and NeuroBlate (Monteris Medical, Winnipeg, Manitoba, Canada), operating at the laser wavelengths of 980 nm and 1064 nm, respectively [35–38].

The tissue optical properties depend upon the laser wavelength, and are introduced in Eq. 9 in terms of the effective attenuation coefficient based on diffusion approximation:

11 $$\mu_{eff} = \sqrt {3\mu_{a} \mu^{\prime}_{s} } ,$$

where *µ*_*a*_ and *µ′*_*s*_ is the absorption and the reduced scattering coefficient of the tissue. Due to the unavailability of experimental data, the absorption coefficient for calf brain tissue at the laser wavelength of 980 and 1064 nm has been assumed to be similar to the porcine brain tissue at the respective wavelengths as has been found to be 0.314 cm^−1^ and 0.1264 cm^−1^, respectively, in [39]. Similarly, the reduced scattering coefficient for calf brain tissue at the laser wavelength of 980 and 1064 nm has been assumed to be similar to the porcine brain tissue as found to be 13.03 cm^−1^ and 14.6 cm^−1^, respectively [39].

The heat flux Eq. 1 is modified by Cattaneo [40] and Vernotte [41] to account for the thermal phase lag (*τ*_*q*_) associated with the time delay between the heat flux and temperature gradient, as:

12 $$q(\vec{r},\;t + \tau_{q} ) = - k\nabla T(\vec{r},\;t),$$

where *τ*_*q*_ is the thermal relaxation time. The constitutive equation thus obtained is known as Cattaneo–Vernotte (C–V), or single-phase-lag (SPL), non-Fourier heat transfer model as given by:

13 $$\tau_{q} \rho c_{eff} \frac{{\partial^{2} T}}{{\partial t^{2} }} + \left( {\rho c_{eff} + \tau_{q} \omega_{b} \rho_{b} c_{b} } \right)\frac{\partial T}{{\partial t}} = k\nabla^{2} T - \omega_{b} \rho_{b} c_{b} (T - T_{b} ) + Q_{m} + Q_{laser} + \tau_{q} \left( {\frac{{\partial Q_{m} }}{\partial t} + \frac{{\partial Q_{laser} }}{\partial t}} \right).$$

The conduction relation between heat flux and temperature gradient was later modified by Tzou [42] to account for the micro-structural interaction and thermal inertia effects, as:

14 $$q(\vec{r},\;t + \tau_{q} ) = - k\nabla T(\vec{r},\;t + \tau_{t} ),$$

where as before, *τ*_*q*_ is the phase lag for the heat flux, *τ*_*t*_ is the phase lag for the temperature gradient, and the constitutive equation obtained incorporating both these phase lags is known as the dual-phase-lag (DPL) non-Fourier heat transfer model given by:

15 $$\tau_{q} \rho c_{eff} \frac{{\partial^{2} T}}{{\partial t^{2} }} + \left( {\rho c_{eff} + \tau_{q} \omega_{b} \rho_{b} c_{b} - \tau_{t} k\nabla^{2} } \right)\frac{\partial T}{{\partial t}} = k\nabla^{2} T - \omega_{b} \rho_{b} c_{b} (T - T_{b} ) + Q_{m} + Q_{laser} + \tau_{q} \left( {\frac{{\partial Q_{m} }}{\partial t} + \frac{{\partial Q_{laser} }}{\partial t}} \right).$$

The above equation is reduced to the SPL non-Fourier model (Eq. 13) for τ_t_ = 0, and Fourier–Pennes’ model (Eq. 2) for τ_q_ = τ_t_ = 0.

The finite element method (FEM) based solver, COMSOL Multiphysics (COMSOL, Inc., Stockholm, Sweden), has been adopted to conduct bioheat transfer analysis of interstitial laser ablation performed on ex vivo brain tissue. The computational domain has been discretized using the COMSOL built-in mesh generator with additional refinements close to the laser fiber applicator. A grid independence study has been conducted to obtain a mesh-independent solution and optimize the computational resources. The optimal number of triangular mesh elements was determined by progressively refining the mesh until the error between the predicted temperature rise at a selected point and the predicted ablation volume was less than 0.5% between two contiguous meshes. The final mesh consists of 18,874 triangular elements. Further, a time step of 0.001 s was defined for solving the heat transfer within the computational domain based on the convergence study of the time step.

### Experiments on Ex Vivo Calf Brain

**Fig. 2:**
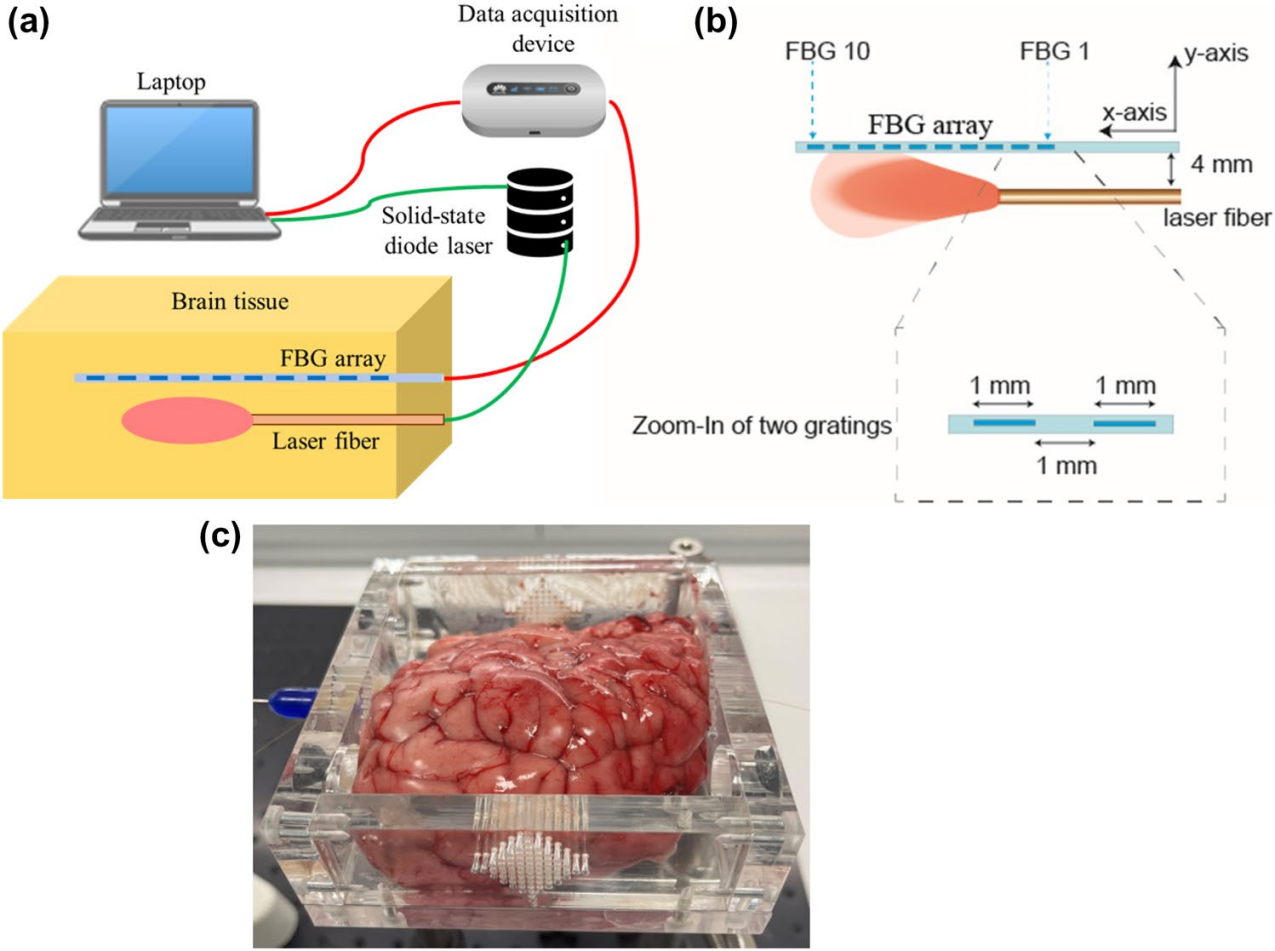
**a** Schematic of the experimental setup of interstitial laser ablation, **b** close-up of the positioning of laser applicator and fiber Bragg grating (FBG) sensor, and **c** photo of the calf brain tissue placed in the plexiglass box having holes at a desired location to maintain the positioning of laser fiber and FBG sensor

To evaluate the fidelity and integrity of the developed model, interstitial laser ablation was performed on freshly excised ex vivo calf brain tissue that was procured from the local abattoir on the same day of the experiment. The tissue was stored in the refrigerator at 4 °C and was kept at room temperature for about 2 h before performing experiments. A 1064 nm diode laser (LuOcean Mini 4, Lumics, Berlin, Germany) was utilized to irradiate the brain tissue. The light coming from the NIR laser working in continuous-wave mode was administered to the brain tissue using flexible quartz optical fiber (300 µm diameter). Figure 2 shows the experimental setup used in this study for performing interstitial laser ablation on ex vivo calf brain tissue.

**Fig. 3:**
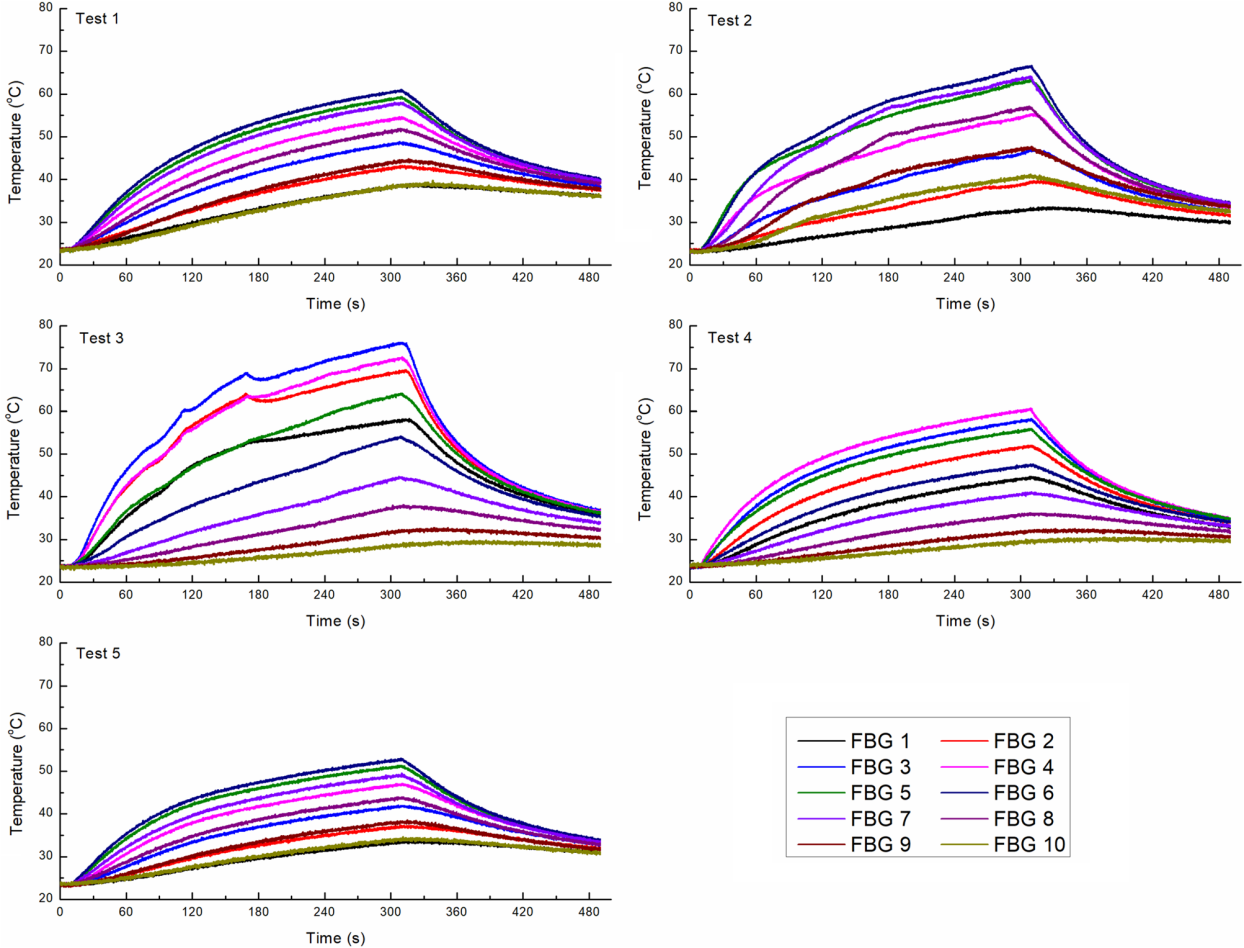
Temperature profile recorded by the FBG array (chain of 10 FBG sensors) placed at a distance of 4 mm parallel from the laser applicator during the interstitial laser ablation of the calf brain tissue

A total of five experiments were performed on the calf brain tissue with a laser power of 2 W delivered for 300 s, with the aim of measuring the brain temperature distribution caused by the interstitial laser ablation. We started temperature measurements at t = 0 s. Motivated by [12, 34], at t = 10 s, the laser power was switched ON, and at t = 310 s, the laser power was turned OFF, and at t = 490 s, measurements were stopped. The real-time temperature was monitored utilizing fiber Bragg grating (FBG) sensors placed at a distance of 4 mm parallel from the laser applicator. Notably, we have used an FBG array (chain of 10 FBG sensors), with each FBG having a 1 mm grating length and a 1 mm distance between grating edges, making the total sensing length ~ 19 mm. A custom-made plexiglass box with holes at desired locations was used to guarantee the accurate positioning of the laser applicator and the sensor. FBG sensors are particularly suited for this application: they are characterized by low thermal conductivity [43], thus reducing the heat transfer between adjacent gratings and are made of non-metallic materials thus avoiding self-heating which in turn causes temperature overestimation [44]. More details about the FBG sensors, measurements, and positioning of the sensors and laser applicators in the ex vivo tissue during interstitial laser ablation can be found in other recent studies from our group, e.g., in [12, 34, 45]. Figure 3 presents the temperature distribution recorded by the chain of 10 FBG sensors placed at a distance of 4 mm from the laser applicator within the calf brain tissue for the five experimental tests. The maximum temperature measured by the FBG sensors placed at 4 mm from the laser applicator tip was compared to the one numerically predicted from the computational model. The experimental temperature is reported as the mean value and standard deviation, calculated considering the results of the five experiments.

## Results

This section will present the results derived from the developed non-Fourier bioheat transfer model of interstitial laser ablation applied to brain tissue. In what follows, we will first systematically investigate the effect of temperature-dependent thermal properties, non-Fourier lags, and blood perfusion rate on the interstitial laser ablation of the ex vivo brain. The developed numerical model will then be validated with the results obtained from experimental laser ablation studies on ex vivo calf brain tissue. Finally, we will perform parametric studies to quantify the effects of input laser power, treatment time, laser fiber diameter, and laser wavelength on the efficacy of the interstitial laser therapy of brain tissue, considering in vivo settings.

### Ex Vivo Model with Experimental Validation

#### Effect of Temperature-Dependent Thermal Properties

**Fig. 4:**
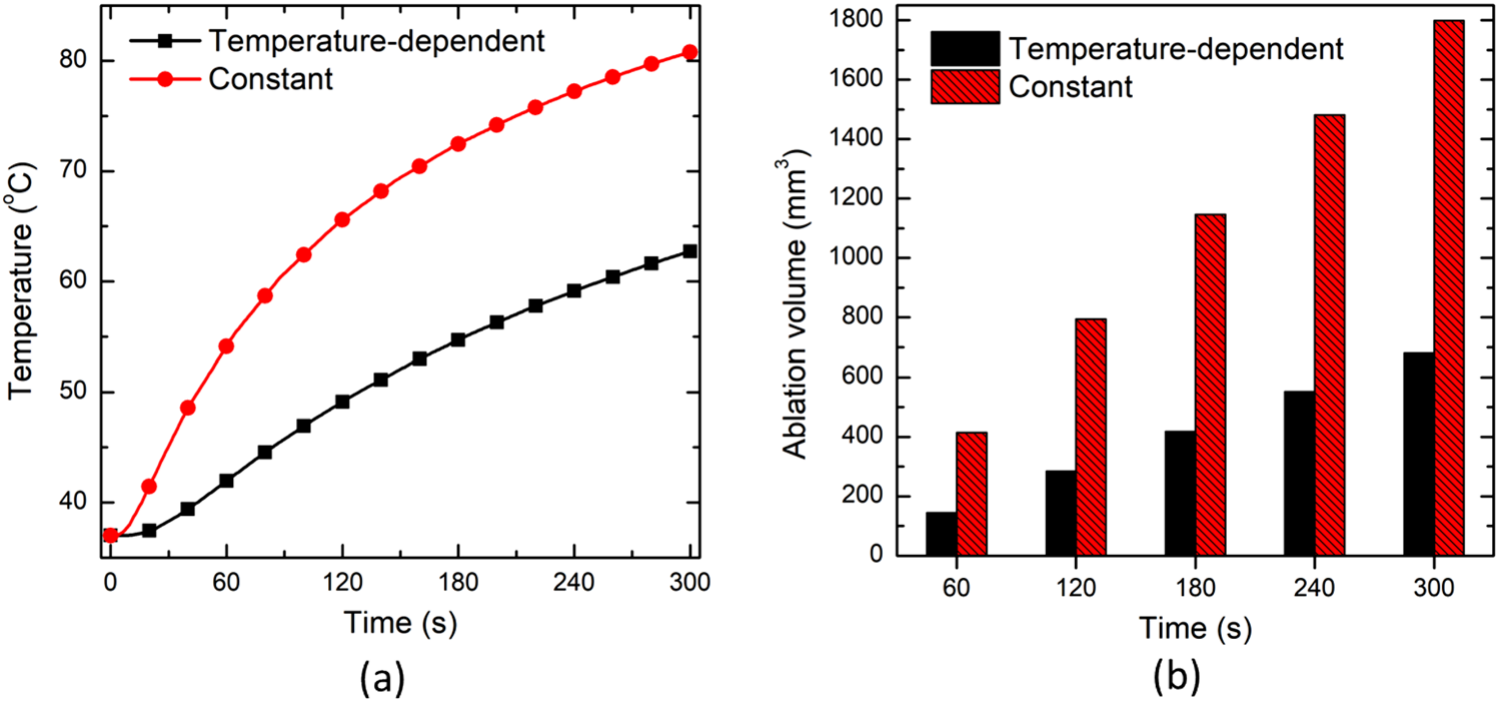
**a** Temperature profile at a point located at 4 mm radially away from the center of the laser fiber tip. **b** Ablation volume obtained by considering constant and variable (temperature-dependent) thermal properties during the interstitial laser ablation of ex vivo brain tissue

Figure 4 presents the result of a comparative analysis conducted to quantify the variation in the predicted temperature distribution (Fig. 4a) and ablation volume (Fig. 4b) during interstitial laser ablation utilizing the bioheat transfer model in ex vivo brain tissue considering constant (@ 22 °C) and non-linear temperature-dependent (variable) thermal properties. The laser power of 2 W was delivered for 300 s through the laser fiber applicator of 300 µm in diameter. The temperature monitoring location has been assumed to be the same as the placement of FBG sensors used in the experimental validation ("Effect of Microvascular Perfusion" section), i.e., at a distance of 4 mm parallel from the laser applicator. Indeed, this setting of laser ablation and temperature monitoring location would be the same throughout the proceeding sections until otherwise stated. Figure 4a depicts that the temperature predicted by considering the temperature-dependent thermal properties is significantly lower than that predicted with constant temperature thermal properties. This variation increases with the increase in treatment time. After 300 s of laser ablation, the temperature predicted by the constant thermal properties model is 28.9% higher than the temperature-dependent thermal properties model prediction. Similarly, the ablation volume predicted by the constant thermal properties model is 164.4% higher than that predicted by the temperature-dependent thermal properties model. The attainment of higher temperature and ablation volume for constant thermal properties can be attributed to the higher value of thermal conductivity leading to faster heat diffusion within the tissue. Notably, these significantly higher variations can be attributed to the fact that constant power laser mode was applied in this study to ablate the tissue, and thus a significant portion of the tissue will be charred corresponding to temperatures greater than 100 °C. Considering the same values of thermal properties for the healthy and charred tissue (or discarding the effects of water vaporization) will lead to erroneous predictions. These deviations would be less in the case where some controlled algorithms (such as temperature feedback-controlled PID controller, and pulsed or ON–OFF cycles power controllers, as highlighted in [12, 34, 46]) were utilized to keep the maximum temperature way below the 100 °C, where the abrupt changes in the thermal characteristics of biological tissue occur due to charring.

#### Effect of Non-Fourier Thermal Relaxation Phase Lags

**Fig. 5:**
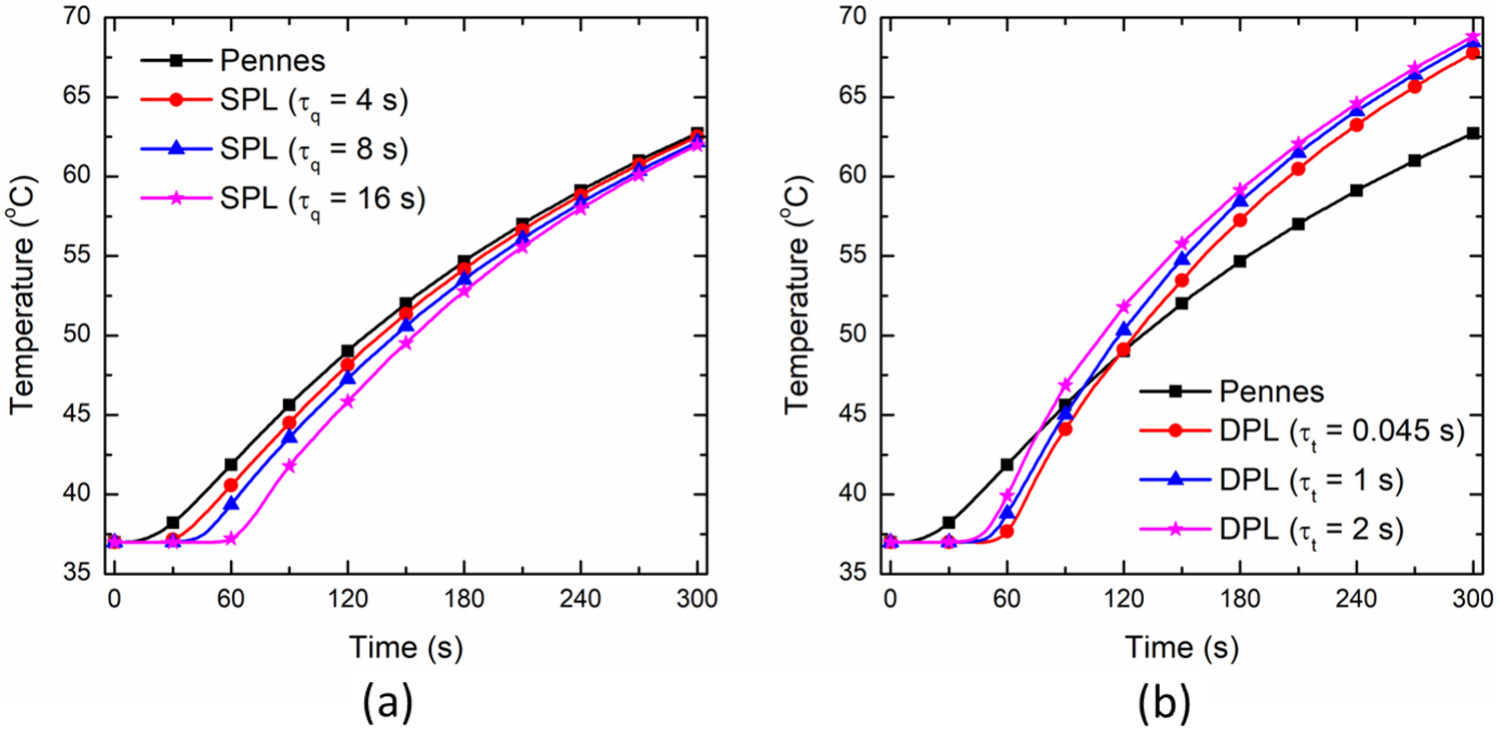
Effect of variation in the magnitude of **a** τ_q_, and **b** τ_t_ on the temperature profile monitored at a location of 4 mm radially away from the center of the laser fiber tip in comparison to the Pennes bioheat transfer model predictions

The effect of variation in τ_q_ on temperature distribution utilizing the SPL non-Fourier bioheat transfer model has been presented in Fig. 5a. As evident, the temperature profile attained with SPL non-Fourier model for different values of τ_q_ is always lower than the one predicted by the Fourier law-based Pennes bioheat transfer model. Initially, for the first 60 s or so, the deviation among the temperature profile predicted with Pennes and SPL models is higher that eventually decreases with the passage of treatment time. Moreover, the predicted temperature profile with the SPL model decreases with an increase in the magnitude of τ_q_. This can be attributed to the fact that the higher the magnitude of τ_q_, the higher would be the energy accumulation. Subsequently, higher vibration characteristics would be induced in the hyperbolic non-Fourier bioheat transfer model of interstitial laser ablation. Further, it has been found that after 60 s, the attained ablation volume within brain tissue are 140.94, 128.38, 120.06, and 109.49 mm^3^ with Pennes, SPL (τ_q_ = 4 s), SPL (τ_q_ = 8 s), and SPL (τ_q_ = 16 s) bioheat transfer models, respectively. After 300 s of laser therapy, the ablation volumes were found to be 678.92, 672.93, 658.13, and 653.23 mm^3^ with Pennes, SPL (τ_q_ = 4 s), SPL (τ_q_ = 8 s), and SPL (τ_q_ = 16 s) models, respectively.

The effect of variation in τ_t_, with fixed τ_q_ = 16 s, on the predicted temperature profile of the DPL non-Fourier model has been shown in Fig. 5b. As depicted in this figure, the temperature profile predicted by the DPL model increases with an increase of the magnitude of τ_t_. This is because the increase in the magnitude of τ_t_ results in lower energy accumulation and, subsequently, more diffusion and diminishing vibration characteristics in response to the elevated temperature attained during laser therapy. Furthermore, as evident from Fig. 5b, the Pennes model prediction of the temperature profile is initially higher than DPL predictions for different values of τ_t_, which overturns completely after a particular time, from where the DPL model temperature predictions are higher than Pennes model predictions. This can be attributed to the fact that initially, the impact of τ_q_ is predominant, resulting in energy accumulation and, thus, lesser thermal diffusion and temperature rise. Afterward, the impact of τ_t_ is higher than τ_q_, resulting in less energy accumulation and more thermal diffusion and temperature rise away from the laser fiber applicator. Such findings are consistent with that reported in [47, 48]. The attained ablation volume with DPL non-Fourier bioheat transfer models after 60 s and 300 s are presented in Table 1.

**Table 1:** Variation of ablation volume attained with non-Fourier heat transfer models post 60 s and 300 s of interstitial laser ablation procedure in the brain tissue

DPL relaxation times	Ablation volume after t = 60 s	Ablation volume after t = 300 s
τ_q_ = 16 s, τ_t_ = 0.045 s	106.44 mm^3^	654.31 mm^3^
τ_q_ = 16 s, τ_t_ = 1 s	118.3 mm^3^	674.96 mm^3^
τ_q_ = 16 s, τ_t_ = 2 s	141.38 m^3^	689.47 m^3^

#### Experimental Validation of Ex Vivo Model

**Fig. 6:**
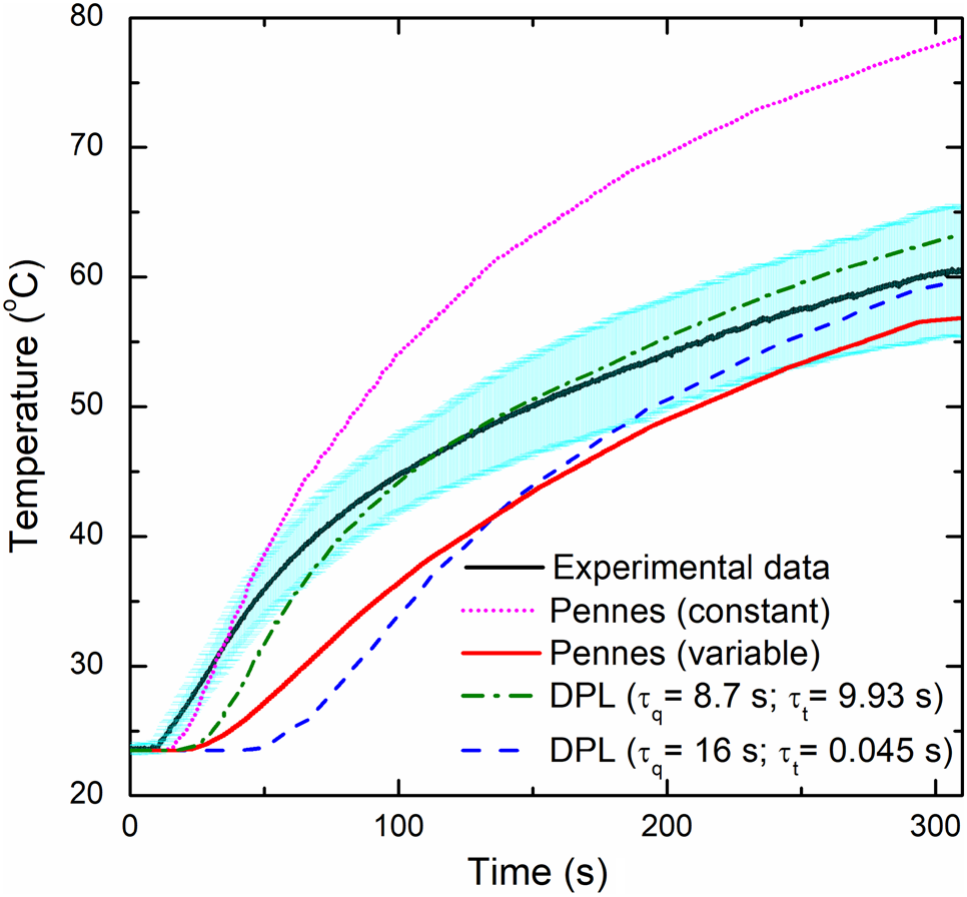
Comparison between the experimentally measured and numerically predicted values of temperature variation with time during interstitial laser ablation of ex vivo calf brain tissue. Here, the black curve represents the mean value of the temperature of experimental findings, and the cyan color represents the standard deviation obtained after five experimental trials

Since ex vivo calf brain tissue does not possess blood perfusion nor metabolic heat generation, these effects have been neglected in the numerical simulation of interstitial laser ablation for validation purposes. In the later section, these terms will be included to report more concrete results translated to the actual human brain tissue. Figure 6 presents the comparison between the experimentally measured temperature from the FBG sensors (placed 4 mm parallel from the laser applicator), and numerically predicted from: (a) the Fourier bioheat transfer model considering constant and temperature-dependent thermal properties of brain tissue; (b) non-Fourier phase lag models (DPL) considering temperature-dependent properties of brain tissue. It is noteworthy to mention that owing to the lack of availability of experimental data for phase lags (τ_q_ and τ_t_) of calf brain tissue, the validation has been done considering two cases of DPL models: (i) τ_q_ = 16 s and τ_t_ = 0.045 s based on previous studies related to laser-assisted photothermal therapy [49, 50], and (ii) τ_q_ = 8.7 s and τ_t_ = 9.93 s based on the average values of numerically predicted non-Fourier lags of brain quantified considering properties of artery, vein and tissue available in literature and reported by Afrin et al. [51]. Figure 6 compares the maximum temperature experimentally measured from the FBG sensors and numerical predicted using Pennes (constant thermal properties), Pennes (temperature-dependent (or variable) thermal properties), DPL (τ_q_ = 16 s, τ_t_ = 0.045 s), and DPL (τ_q_ = 8.7 s, τ_t_ = 9.93 s). As evident from Fig. 6, the numerical prediction of the temporal temperature profile with the Fourier (Pennes) model with constant thermal properties (pink curve) is significantly overestimated as compared to the experimental findings. Furthermore, the numerical predictions obtained with the Pennes model with temperature-dependent properties (red curve) are compatible with the band of the experimental values but underestimate the mean value (black curve) in the heating zone. Among Pennes and DPL model predictions with temperature-dependent thermal properties, the maximum temperature predicted with the DPL model with τ_q_ = 8.7 s and τ_t_ = 9.93 s is more close to the experimental observations. Further investigations are warranted to refine the DPL model predictions by accurately quantifying the thermal phase lag values for different tissues of interest. Moreover, it is noteworthy to mention that the deviation between experimental and numerical findings could also be linked to the rate of degradation and tissue consistency across the samples that have not been accounted in the present study. Lastly, the variability on the measured temperature can be related to the intrinsic variability of the different organs and regions of the brain used to perform the experimental validation, as well as to the influence of the sensors’ resolution on the measurement of temperature in presence of a high thermal gradient, as has been reported by Morra et al. [43].

#### Effect of Microvascular Perfusion

**Fig. 7:**
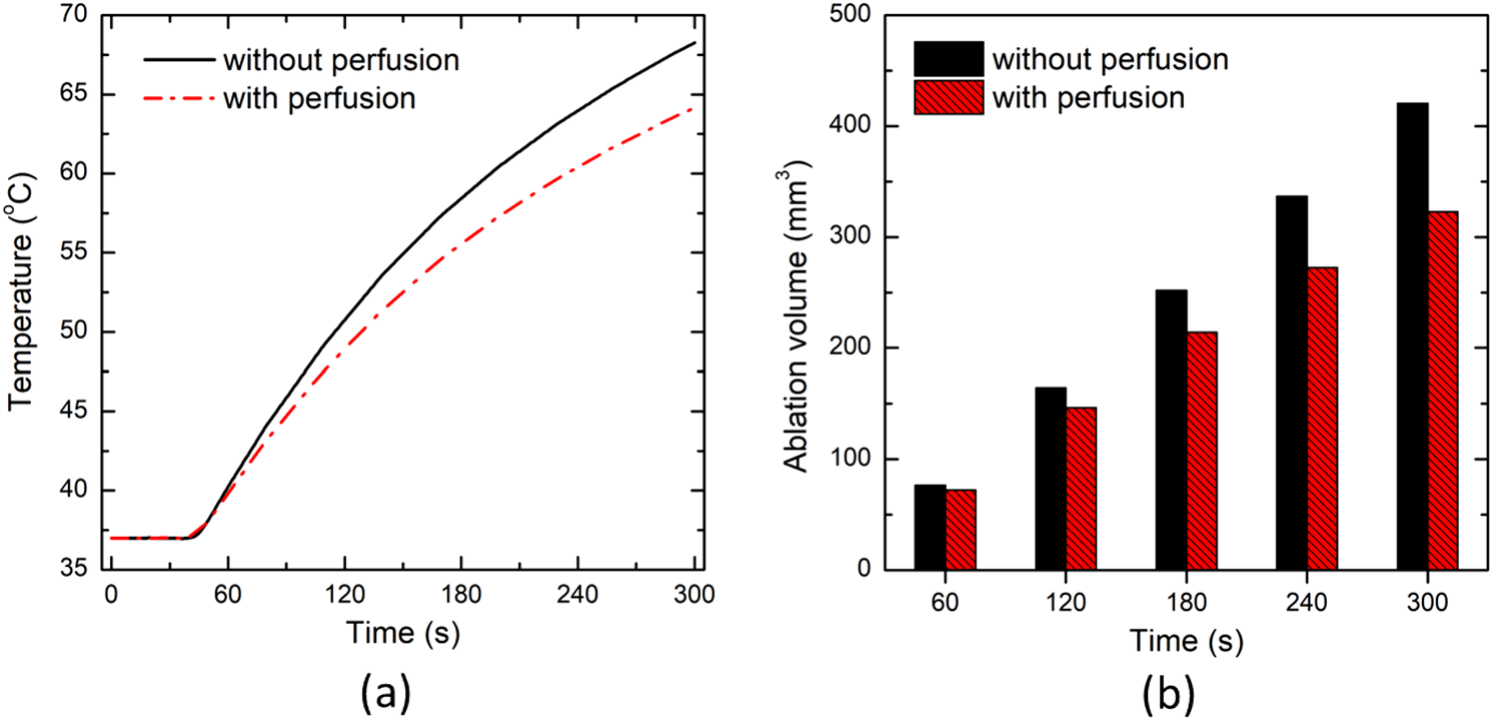
**a** Temperature profile predicted at a location of 4 mm radially away from the center of the laser fiber tip. **b** Ablation volume under non-perfused (i.e., without microvascular perfusion) and perfused (i.e. considering microvascular perfusion) settings of brain tissue during laser ablation

In this section, we will extend the non-Fourier non-perfused brain tissue model, including temperature-dependent thermal properties for interstitial laser ablation as reported in the above two sections, incorporating the microvascular blood perfusion as presented in Eq. 7. Motivated by the more reasonable DPL model predictions in comparison to the experimental findings for non-Fourier lags values of τ_q_ = 8.7 s and τ_t_ = 9.93 s, we will assume these values throughout this study. Figure 7 presents the influence of the blood perfusion rate of the brain tissue on the temperature distribution and ablation volume predictions. As expected, the predictions of the temperature distribution for the non-perfused brain model is higher than the perfused model due to the additional heat-sink effect caused by the blood perfusion in perfused brain model. The deviation in the predicted temperature profile increases with the increase in interstitial laser ablation treatment time, as evident from Fig. 7a. After 300 s, the deviation in the temperature predicted in ex vivo and in vivo brain tissue has been found to be 6.04%. The comparison of ablation volume predicted without blood perfusion and with blood perfusion at different treatment times has been presented in Fig. 7b. Again, as a consequence of convective heat transfer induced due to the blood perfusion, ablation volume obtained within in vivo brain tissue is lower as compared to ex vivo brain. In other words, the presence of the heat sink effect in the perfused brain tissue will result in more input requirements of laser energy to ablate the same amount of non-perfused brain tissue. The ablation volume attained after 60 s of laser therapy for non-perfused and perfused tissue has been found to be 76.34 mm^3^ and 72.31 mm^3^, respectively. After 300 s, the attained ablation volume increases to 420.61 mm^3^ and 322.84 mm^3^ for non-perfused and perfused brain tissues, respectively.

### In Vivo Model: Effect of Input Laser Power, Laser Fiber Applicator Diameter, and Wavelength

**Fig. 8:**
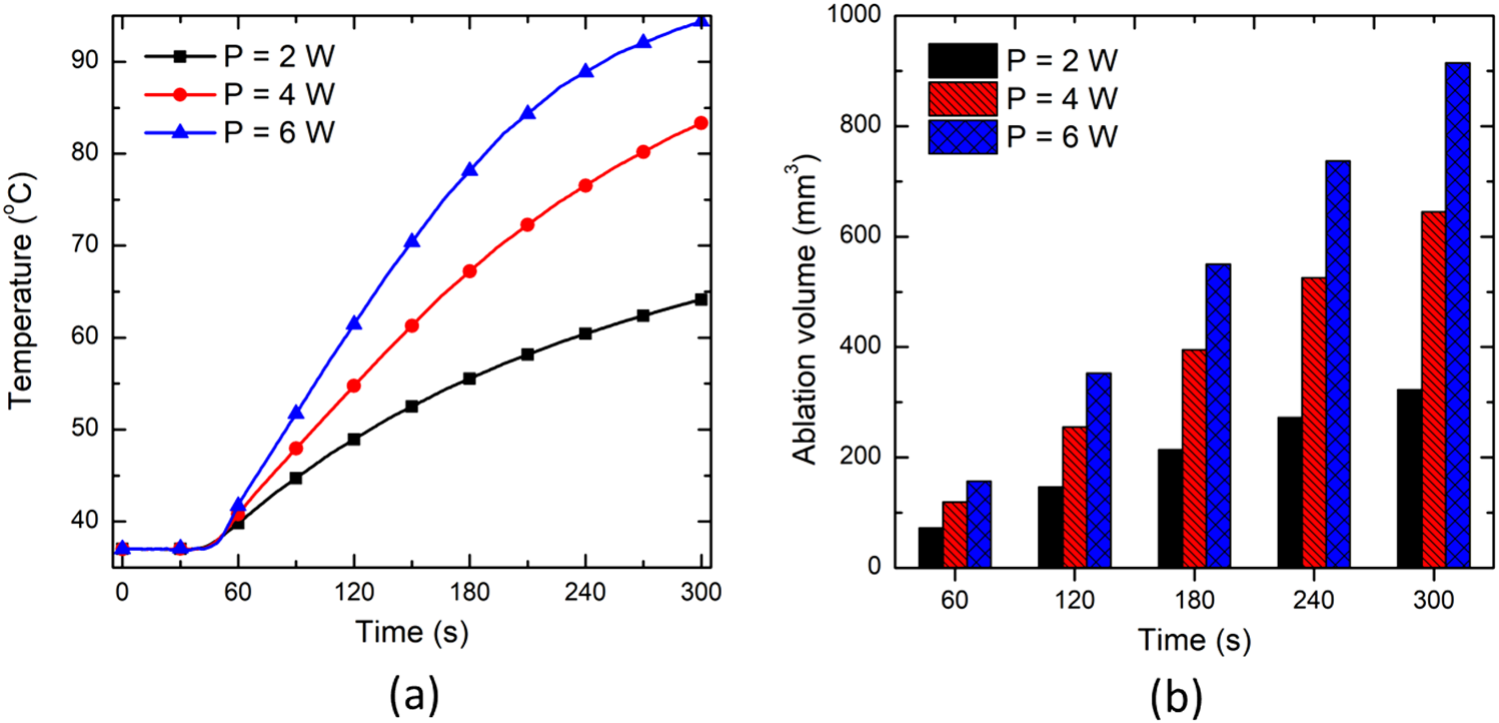
Effect of input laser power on **a** the temperature profile predicted at a location of 4 mm radially away from the center of the laser fiber tip, and **b** the ablation volume during interstitial laser ablation

This section will report the results of the parametric analysis conducted on the DPL non-Fourier bioheat transfer model of perfused brain tissue incorporating temperature-dependent thermal properties as reported in previous sections, considering τ_q_ = 8.7 s, and τ_t_ = 9.93 s. Here, laser power values of 2–6 W have been considered with an irradiation time of 300 s. Further, the diameter of the laser fiber has been varied in the small range of 300 µm to 800 µm, so as to maintain the relative merit of the lower thickness of the laser applicator in comparison to the applicators of other thermal ablative modalities, viz, radiofrequency, microwave, and cryoablation. Figure 8a presents the temperature profile obtained at a location of 4 mm radially away from the center of the laser fiber tip for different values of input laser power. This figure shows that an increase in input laser power results in a corresponding increase in the predicted temperature. However, the relative jump in temperature attained by increasing power from 2 to 4 W is not the same as obtained by increasing power from 4 to 6 W. For example, the increase in temperature obtained after 300 s of treatment time is 29.9% as input laser power increases from 2 to 4 W. It remains at just 13.28% for an input power increase from 4 to 6 W. This effect can be attributed to the charring phenomena (water vaporization) that result in a significant decline in the thermal properties of the tissue for temperature greater than 100 °C. Thereby acting as a barrier and restricting the efficient heat conduction within the tissue to more peripheral areas away from the applicator.

**Fig. 9:**
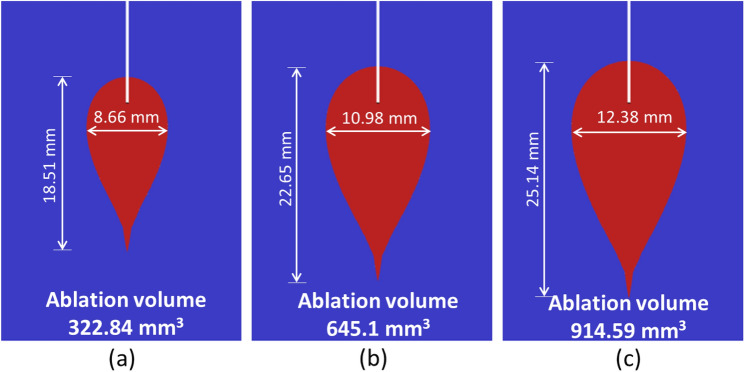
Schematic of the ablation volume shapes attained after 300 s of interstitial laser ablation in brain tissue with **a** 2 W, **b** 4 W, and **c** 6 W of input laser power

Figure 8b presents the evolution of ablation volume with treatment time for different values of input laser power. As depicted in Fig. 8b, the ablation volume increases with the increase in both treatment time and power. The ablation volume attained with 6 W of laser power is significantly higher as compared to that attained with 2 W. Thus, it becomes absolutely essential to select an optimal combination of power and treatment time for ablating a desired volume of tumorous tissue (sparing healthy tissue) during interstitial laser ablation. Figure 9 presents the pictorial representation of ablation volume attained in the in vivo brain tissue for different laser power values after 300 s of therapy. The shapes of the ablative region presented here are consistent with those reported in previous literature, as by Saccomandi et al. [16].

**Fig. 10:**
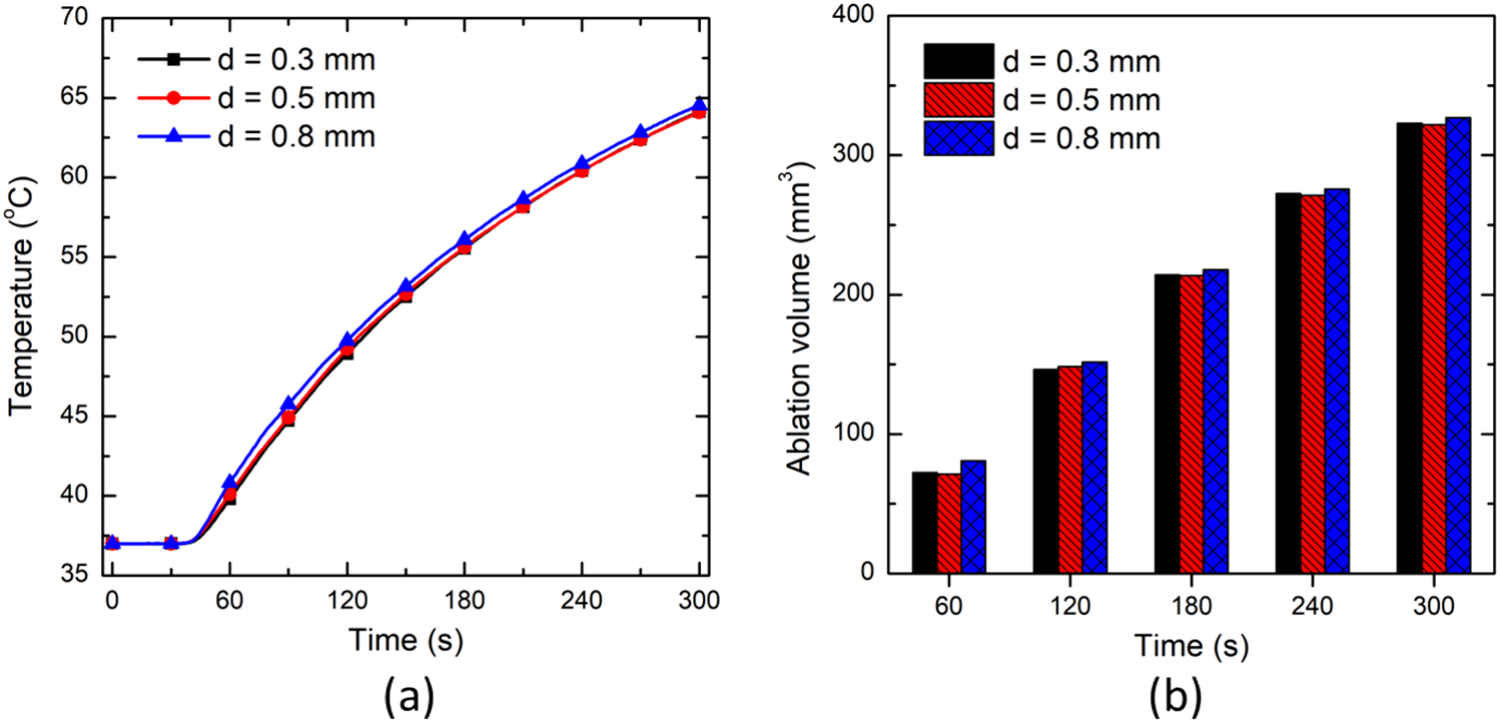
Effect of laser fiber applicator diameter on **a** the temperature profile predicted at a location of 4 mm radially away from the center of the laser fiber tip, and **b** the ablation volume during interstitial laser ablation

Figure 10 presents the variation in temperature distribution and ablation volume for different laser fiber applicator diameter values. This figure shows insignificant variations in the predicted temperature profile and ablation volumes post 300 s of interstitial laser ablative procedure of in vivo brain tissue. The variations in the temperature profile and ablation volume may change for higher values of the laser fiber diameter. But as mentioned before, we want to keep the maximum diameter of the laser fiber applicator to less than 1 mm to preserve the advantage of lower applicator thickness of interstitial laser ablation over other thermal ablative therapies. Therefore, the small variations in the laser fiber diameter have minimal impact on the treatment outcomes of the interstitial laser therapy of brain tissue. Moreover, the maximum intensity at the center of the laser fiber decreases with an increase in the radius of the laser fiber for a constant value of power (refer to Eq. 10). The maximum intensity at the center of the laser beam for the diameters of 300 µm, 500 µm, and 800 µm has been found to be 12 × 10^7^ W/m^2^, 4.5 × 10^7^ W/m^2^ and 1.6 × 10^7^ W/m^2^, respectively. Thus, highlighting more focused energy deposition within the biological tissue for lower laser fiber applicator diameter values.

**Fig. 11:**
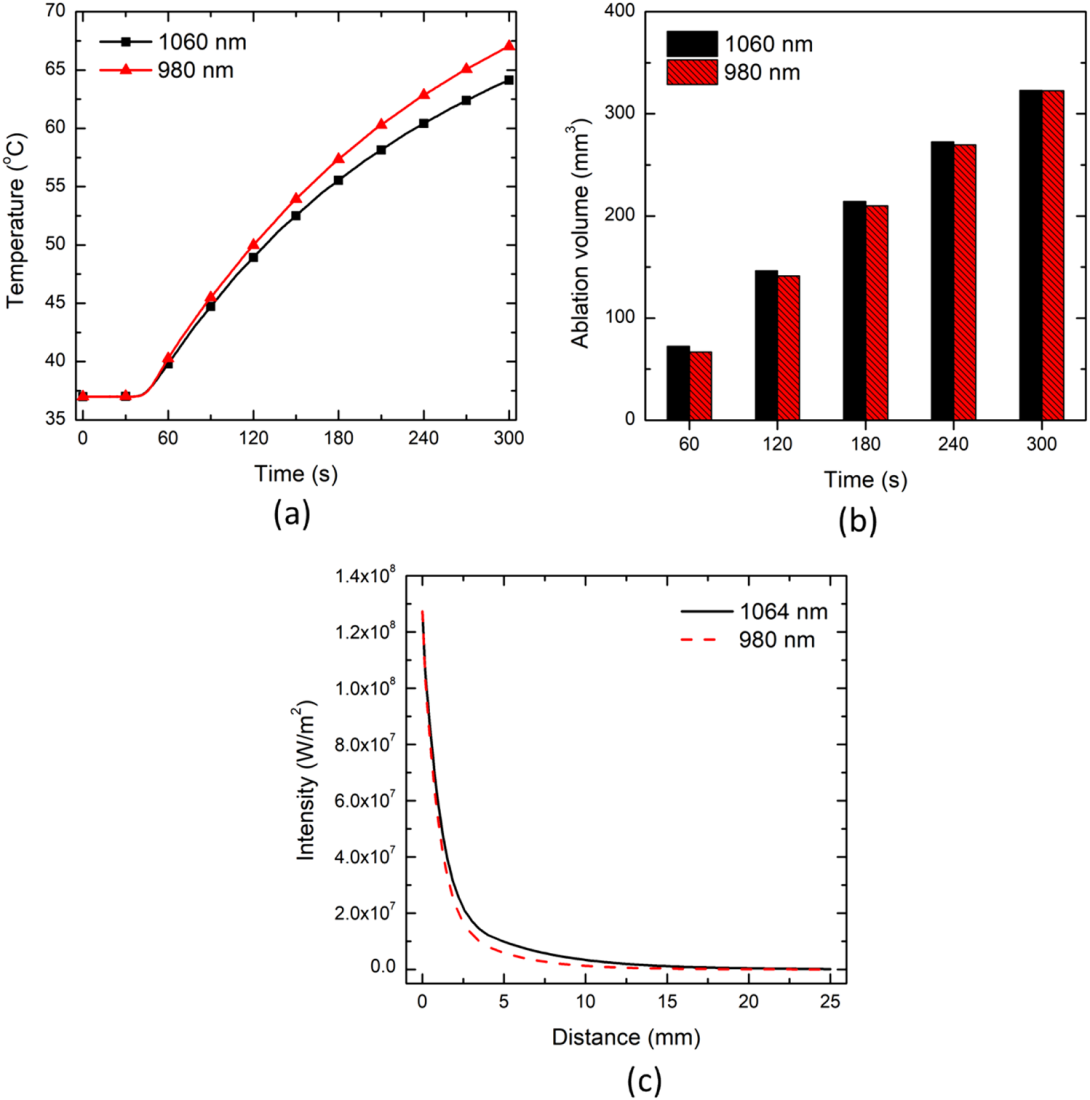
Effect of laser wavelength on **a** the temperature profile predicted at a location of 4 mm radially away from the center of the laser fiber tip, **b** the ablation volume, and **c** the irradiation profile

Figure 11 presents the effect of laser wavelength on the temperature distribution, ablation volume, and irradiation profile for 980 nm and 1064 nm wavelengths. Notably, the selected two wavelengths are consistent with the laser wavelengths of the U.S. FDA-approved interstitial laser ablation devices. It can be seen from Fig. 11 that the temperature profile with the laser wavelength of 980 nm is higher than the one obtained with 1064 nm. This can be attributed to the variations in absorption and scattering coefficient of tissue for different wavelengths, as has been recently characterized Mosca et al. and reported in [39] for calf brain tissue. The absorption coefficient for calf brain tissue at the laser wavelengths of 980 and 1064 nm has been found to be 0.314 cm^−1^ and 0.1264 cm^−1^, respectively [39]. By virtue of this variation in absorption coefficients, the thermal diffusion would spread farther due to higher temperature rise for a light with relatively higher absorption. This has also been depicted in Fig. 11c, which presents the intensity profile predicted from the center of the laser fiber along the depth of the tissue in the forward direction. Both the maximum intensity and the depth of penetration are higher for the 980 nm laser wavelength when compared to the 1064 nm wavelength. From ablation volume perspective, there is negligible variation at the end of 300 s for both the selected laser wavelengths. It is worth mentioning that slightly higher prediction from 60 to 240 s durations for laser wavelength of 1064 nm can be linked to the charring phenomena and may need further investigation. Thus, highlighting the importance of the proper selection of wavelength for enhancing the efficacy of interstitial laser ablation based on the optical properties of the biological tissues under consideration.

## Discussion

The accuracy of temperature prediction with the governing equations of different bioheat transfer models presented in "Materials and Methods" section tremendously depends on the thermal properties of the tissue under consideration. It is a common practice to treat the thermal properties of the tissue (viz., thermal conductivity, volumetric heat capacity, and thermal diffusivity) as constant, generally at room temperature, during computational modeling of thermal therapies [10, 32, 33, 36, 47, 49, 52, 53]. This simplified assumption is also considered due to the lack of experimental data available in the literature related to the thermal characterization of biological tissues in the temperature range applicable for thermal therapies [54]. Thus, we conducted a comparative analysis to highlight the differences in the predicted temperature distribution considering the brain tissue's constant and experimentally derived variable (temperature-dependent) thermal properties during interstitial laser ablation as presented in Fig. 4. Importantly, our group has acquired the thermal properties of the ex vivo calf brain tissue in the temperature range from 22 to around 97 °C, as has been reported by Mohammadi et al. [30]. It was found that the thermal properties of the brain have a non-linear relationship with temperature. The thermal properties (i.e. thermal conductivity and volumetric heat capacity) were almost constant until 60 to 70 °C, gradually rising until 92 °C, and then abruptly increasing from 92 to 97 °C. Furthermore, it is well known that water vaporization occurs when the temperature of biological tissue exceeds 100 °C [7, 55]. This phase change process can be implemented utilizing the apparent heat capacity method framework presented in [26, 29]. We combined the experimental predictions of the ex vivo calf brain tissue reported Mohammadi et al. [30] till 100 °C and the water vaporization model to account for phase transformation as presented in Eqs. 3–6. It has been found that significant variations are prevalent in the temperature and ablation volume predictions highlighting the importance of either considering temperature-dependent thermal properties in the numerical model or incorporating ways to mitigate the attainment of vaporization temperature during interstitial laser ablation of brain tissue.

Considering non-Fourier behavior is crucial to more accurately analyze the heat transfer in biological tissues. Unlike the Fourier law-based Pennes model, these non-Fourier models assume a finite speed of heat propagation within biological tissues, i.e., a lagging behavior between heat flux and the temperature gradient. Several models have been proposed to account for this non-Fourier lag by modifying the Pennes bioheat transfer model [27, 33, 47–49, 56–59]. Among these, the SPL model developed by Cattaneo [40] and Vernotte [41], and the DPL model developed by Tzou [42] are the most common. The underlying difference between the SPL and DPL non-Fourier models is that the SPL model considers only one phase lag associated with heat flux, τ_q_. While the DPL model also accounts for another phase lag associated with temperature gradient, τ_t_. Unfortunately, the accurate characterization of the thermal phase lags in biological tissue is highly complex, and thus it is an uninvestigated problem. There is enormous variability in the phase lag values reported in the literature [7]. Therefore, owing to the lack of specific value for the phase lags, we have conducted parametric studies to evaluate the influence of the phase lags on temperature distribution and ablation volume during interstitial laser ablation of ex vivo brain tissue, considering temperature-dependent thermal properties, as presented in Fig. 5. The value of τ_q_ has been varied in the range of 4–16 s, and τ_t_ has been altered in the range of 0.045-2 s. Comparing Pennes, SPL (τ_q_ = 16 s), and DPL (τ_q_ = 16 s, τ_t_ = 0.045 s) bioheat transfer models, the Pennes model predicted 28.72% and 32.41% higher ablation volume after 60 s of laser therapy as compared to SPL and DPL models, respectively. After 300 s, the ablation volume predicted by the Pennes model was just 8.52% and 3.76% higher when compared to SPL and DPL non-Fourier models, respectively. Thus, these results indicate that for 300 s laser irradiation, the non-Fourier effects are small, but that they are more significant for shorter periods of irradiation.

We have extended the developed non-Fourier temperature-dependent thermal properties model of ex vivo brain tissue for interstitial laser ablation to account for the microvascular blood perfusion as well. It has been found that the consideration of microvascular blood perfusion would result in significant deviations in temperature distribution and ablation volume considering non-perfused brain tissue, as presented in Fig. 7. Thus, clearly highlighting the importance of accounting for blood perfusion in the computational model for the accurate prediction of the treatment outcomes during interstitial laser ablation. Laser power, ablation time, the diameter of the laser fiber applicator, and laser wavelength are the most critical input parameters that influence the efficacy of interstitial laser ablation. Thus, we have conducted parametric studies to quantify their impacts on the treatment outcomes of laser therapy in the brain, the results of which are presented in Figs. 8, 9, 10 and 11. These parametric analysis results would assist clinical practitioners in optimizing laser therapy for attaining safe and effective treatment, precisely localizing the therapy and minimizing the damage to surrounding healthy tissue.

One of the major limitations of this study is the consideration of homogeneous brain tissue (without tumor) during interstitial laser ablation. However, this has been done intentionally to have consistency with the ex vivo experimental studies reported in this work, as well as most of the experimental studies available in the literature. We believe the development of the numerical model in its current form will allow for the direct comparison of laser therapy in homogeneous tissues. Albeit, the experimental studies were performed on ex vivo brain tissue, we have extended the model to also account for in vivo settings by incorporating the blood perfusion term. Furthermore, we have computed the ablation volume with an isothermal approach instead of a more accurate Arrhenius equation, owing to the lack of experimental data on the brain tissue's frequency factor and activation energy. Importantly, we have used the isotherm of 60 °C instead of 50 °C, which provides much closer results to the Arrhenius model [60]. Also, the optical properties included in this study are considered to be constant (independent of the tissue temperature history), again owing to the lack of experimental data related to temperature-dependent optical properties of brain tissue available in the literature. Future studies are required to investigate the progressive change in the optical properties of tissue with tissue temperature history and the related tissue thermal damage, in order to mitigate these limitations and attain a more robust model for accurate prediction of the brain's temperature distribution and ablation volume during interstitial laser ablation. Thus, providing *a priori* information for better treatment planning and optimization, and warranting the overall success of the therapy. Significant research efforts of our group are currently focused on addressing some of the above-mentioned limitations. Moreover, our group is also working on quantifying the phase lags for different tissue of interest through the experimental setup reported in this work to enhance our understanding about these non-Fourier phenomena, so as to reach a stage where these numerical models of interstitial laser ablation can be readily integrated into the clinical workflow for different tissues and higher prediction efficacy.

In conclusion, this work presents the development of a non-Fourier bioheat transfer model for a more accurate analysis of heat transfer within the brain tissue during interstitial laser ablation. Importantly, we have considered the temperature-dependent thermal properties acquired from conducting experiments on ex vivo brain tissues. The developed model predictions were found to be in satisfactory agreement with the real-time temperature distribution obtained with the FBG sensors during experimental studies of interstitial laser ablation performed on ex vivo calf brain tissues. The results of the parametric analysis conducted to evaluate the influence of laser power, treatment time, laser fiber diameter, laser wavelength, and non-Fourier phase lags on the efficacy of interstitial laser ablation, could significantly assist in the precise assessment of the optimum setting for pre-clinical and clinical laser therapy treatments. We expect that the future development of the model incorporating the actual tumor shape and size, and temperature-dependent thermal and optical properties of the embedded tumor will eventually result in the clinical translation of this model under patient-specific settings.
